# A prospective study evaluating the integration of a multifaceted evidence-based medicine curriculum into early years in an undergraduate medical school

**DOI:** 10.1186/s12909-020-02140-2

**Published:** 2020-08-24

**Authors:** B. Kumaravel, H. Jenkins, S. Chepkin, S. Kirisnathas, J. Hearn, C. J. Stocker, S. Petersen

**Affiliations:** 1 University of Buckingham Medical School, Hunter Street, Buckingham, MK18 1EG UK; 2 Thames Valley Deanery, Oxford, UK; 3 East and North Hertfordshire Clinical Commissioning Group, Welwyn Garden City, UK; 4 Manchester Metropolitan University, Manchester, UK

**Keywords:** Evidence-based medicine, EBM, Competency, Undergraduate medical education, Fresno test, Knowledge, Perceptions

## Abstract

**Background:**

The importance of ensuring medical students are equipped with the skills to be able to practice evidence-based medicine (EBM) has been increasingly recognized in recent years. However, there is limited information on an effective EBM curriculum for undergraduate medical schools. This study aims to test the feasibility of integrating a multifaceted EBM curriculum in the early years of an undergraduate medical school. This was subsequently evaluated using the validated Fresno test and students’ self-reported knowledge and attitudes as they progressed through the curriculum.

**Methods:**

EBM was integrated horizontally and vertically into the curriculum into the first 2 years of undergraduate medical school. First year medical students were recruited to participate in the study. The 212-point Fresno test was administered along with a locally developed questionnaire at baseline before EBM teaching in year one and at the end of EBM teaching in year two.

**Results:**

Thirty-one students participated at baseline and 55 students participated at the end of second year EBM teaching. For the 18 students who completed the Fresno at both time points, the average score increased by 38.7 marks (*p < 0.001*) after EBM teaching. Students felt confident in formulating clinical questions and in critically appraising journal articles after EBM teaching. EBM was perceived to be important to their future practice as a doctor and for improving patient outcomes at both time points.

**Conclusions:**

It has been feasible to integrate a multifaceted, EBM curriculum from the first year of an undergraduate medical program. Early evaluation of the curriculum using the Fresno test has shown a significant increase in students’ EBM knowledge. The curriculum also demonstrated an increase in students’ perceptions of the clinical relevance of EBM in their developing practice.

## Background

Evidence-Based Medicine (EBM) is the triangulation of best available evidence, clinical expertise and the patients’ predicament and preferences before application to clinical decision making [1]. EBM involves five steps- asking the right question; acquiring evidence; appraising evidence; applying to clinical decisions and assessing the performance in the first four steps [2]. The importance of ensuring medical students are fully equipped with the skills to be able to practice EBM has been increasingly recognised in recent years [3, 4]. In order to ensure future medical professionals are better equipped with lifelong skills for evidence-based medicine, it is best to integrate EBM teaching into undergraduate medical curriculum. In its 2018 version of Outcomes for Graduates, the General Medical Council (GMC) stipulates medical graduates should be able to ‘*apply scientific method and approaches to medical research and integrate these with a range of sources of information used to make decisions for care’* (https://www.gmc-uk.org/-/media/documents/dc11326-outcomes-for-graduates-2018_pdf-75040796.pdf).

However, research suggests that only a minority of health professionals regularly apply such an evidence-based approach in their professional practice. Many are deterred by a lack of knowledge of EBM, the required skills and personal time [5]. A study that reviewed EBM teaching in UK medical schools found that students were not taught the skill of applying EBM to real clinical practice and that there was an overreliance on didactic classroom teaching in undergraduate curricula [6]. To aid medical educators, a standardized set of core EBM competencies has been suggested which provides a contemporary set of core competencies to inform curriculum development and benchmarking teaching [7].

Historically, most EBM courses have been taught as short courses, seminars or workshops [8]. It is suggested that EBM teaching should move from classrooms into clinical practice and that multi-faceted, clinically integrated approaches with assessments could be more effective models for teaching EBM [9, 10]. Indeed, this has been demonstrated in a US medical school, where teaching EBM as a longitudinal theme across the medical curriculum rather than standalone courses has been shown to be effective [11]. Clinically integrated teaching of EBM is further likely to bring about a change in skills, attitudes and behaviour [12]. In recent years, different methods of integrating EBM into the undergraduate medical curriculum have been tested and shown to improve EBM competence, knowledge and skills [13, 14].

Besides methods for clinically integrating EBM teaching into undergraduate medical schools, there is increasing guidance for medical educators to evaluate the effectiveness of their educational interventions [10]. To support this, many validated tools are available to evaluate EBM educational interventions [4, 15]. One of these validated tools- the Fresno test of competence in EBM was initially developed to test EBM competencies in family medicine residents [16]. It has since been extensively tested and validated in various other settings. Thomas et al. found that the Fresno test was the only test of EBM competence that had the full set of validity and reliability measures reported [17]. Despite the availability of tools to evaluate EBM teaching, most evidence-based practice educational interventions still do not use high quality tools to measure the outcomes [4].

The University of Buckingham Medical School (UBMS) is a relatively new medical school, with the first intake of students in January 2015. EBM was initially taught as part of the Public Health unit in year one with mostly didactic lectures with small group discussions over 12 weeks. However, students’ feedback at the end of year one EBM teaching suggested that while they grasped the principles, they struggled to understand the practical and clinical relevance of EBM. We have since revised the EBM curriculum- EBM teaching has now been integrated both vertically and horizontally into the undergraduate curriculum and is systematically represented in all assessments. The aim of this study is to evaluate the feasibility and effectiveness of the new EBM curriculum in the first 2 years of the Bachelor of Medicine and Bachelor of Surgery (MBChB) course as assessed by students’ attitudes towards EBM and their competency in the Fresno test.

## Methods

### Revised EBM curriculum

Phase I is the first 2 years of the course when students are mostly taught biomedical sciences with some patient interactions. Phase II is the next two and a half years when students are in clinical placements in hospitals and primary care. In 2015, the medical school had EBM taught in just year one as a standalone 12-week unit, without integration into other parts of the curriculum. Teaching in the 12-week unit involved didactic lectures with minimal interaction with students followed by small group work. The small group tasks focused on learning statistics without clinical context. At the end of the 12-week teaching, students’ feedback suggested that while they understood the concepts, they struggled to see the clinical relevance of EBM concepts. Following this feedback, the EBM lead carried out a literature review, discussed with peers in other medical schools and revised the EBM curriculum in 2016.

**Fig. 1:**
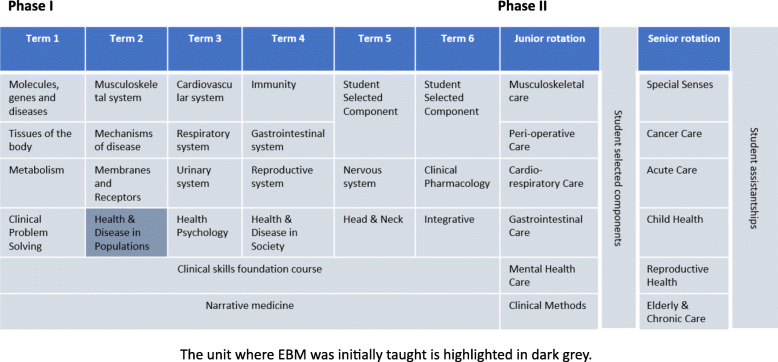
EBM curriculum in MBChB course in UBMS in 2015

**Fig. 2:**
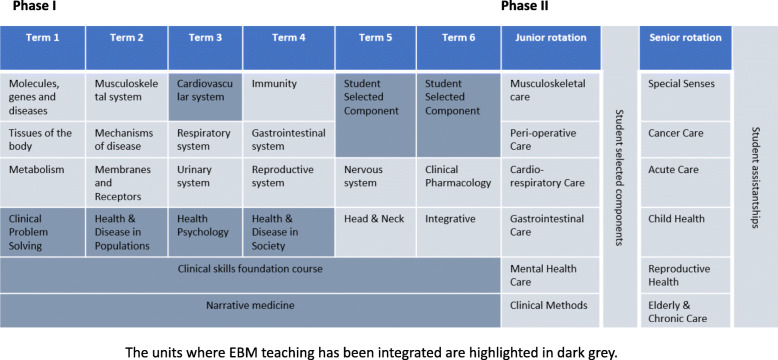
Revised EBM curriculum in MBChB course in UBMS in 2017

In the revised curriculum, EBM teaching is no longer restricted to the 12 weeks unit in year one. The relevance of EBM to other units in Phase I is highlighted by integrating it into teaching and assessments into other units such as Health Psychology and Health and Disease in Society. In addition, when students are taught about clinical skills, clinical problem solving or are asked to write a narrative summary of their patients, they are prompted to think about the evidence base and apply it to clinical reasoning. The original EBM curriculum in 2015 and the revised EBM curriculum in 2017 are shown in detail in Figs. 1 and 2 respectively.

All EBM teaching is delivered by educators who are trained in EBM, including librarians, public health specialists and junior doctors. Teaching starts in term two in year one during the Health and Diseases in Population unit, with an introduction to the five steps of EBM, followed by literature searching, the various epidemiological study designs and critical appraisal. In term three, EBM has been integrated into the Health Psychology Unit, where students learn how to do literature searching using Psych Info, and the Cardiorespiratory Diseases Unit, where the application of EBM to cardiorespiratory disease management is demonstrated. In year two, EBM has been integrated into the Health and Diseases in Society unit in term four, where screening, health economics, evidence based clinical commissioning, literature reviews, and social sciences research methods are taught. Table 1 shows the topics covered in the revised EBM curriculum in Phase I.

**Table 1:**
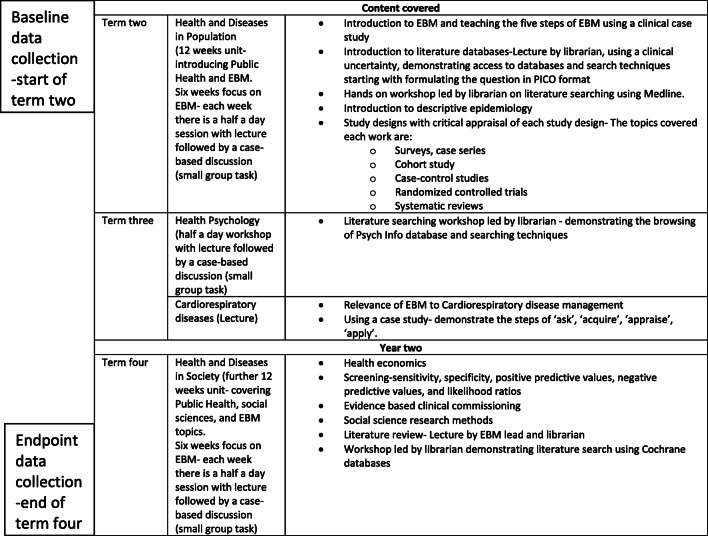
Topics covered in the EBM curriculum in Phase I (along with the two data collection points for this study)

Several elements of the teaching were revised based on pedagogical principles to make teaching more accessible and engaging. Blended teaching methods have been implemented involving the integration of online and face to face teaching activities, as these have been demonstrated to promote engagement [18]. Lectures are now interactive and enhanced with technology, such as audience polls, YouTube videos and Ted talks. Recorded lectures are uploaded onto the virtual learning environment (Moodle), so students can listen to the lectures again in their own time to enhance their learning. Teaching is learner-centered with discussions around visual prompts in lectures and online quizzes. A comparison of teaching methods used in 2015 and 2017 is shown in Table 2.

**Table 2:** Teaching methods in 2015 and 2017

EBM teaching method in 2015	EBM teaching method in 2017
Standalone module over 12 weeks in first year	Integrated vertically and horizontally into the MBChB course
Didactic teaching	Interactive teaching
Focus on learning from textbooks and lectures	Technology enhanced learning/ blended learning methods integrated-- using videos, audios, online quizzes, TED talks and recorded lectures
Traditional model-Class rooms with a traditional style of instruction, using a lecture style followed by students working in small groups on an application task designed by the lecturer	Flipped classrooms introduced-video lessons, online collaboration discussions, research using online databases, knowledge enhancement using peer teaching and clinical problem solving
Small group discussions- focused on learning statistics	Small group discussion -focus on clinical case-based learning
Linear (modular) curriculum	Spiral curriculum-as students progress through the course, they learn the different steps of EBM and each time the previous steps are reinforced.

Small group tasks are based on clinical vignettes, so students engage in case-based discussions and learn to apply EBM principles to clinical vignettes. Students are taught how to ask clinical questions in Patient, Intervention, Control and Outcome (PICO) format; search for the best evidence to answer their question; and critically appraise the validity and reliability of evidence before applying their findings to clinical practice.

**Fig. 3:**
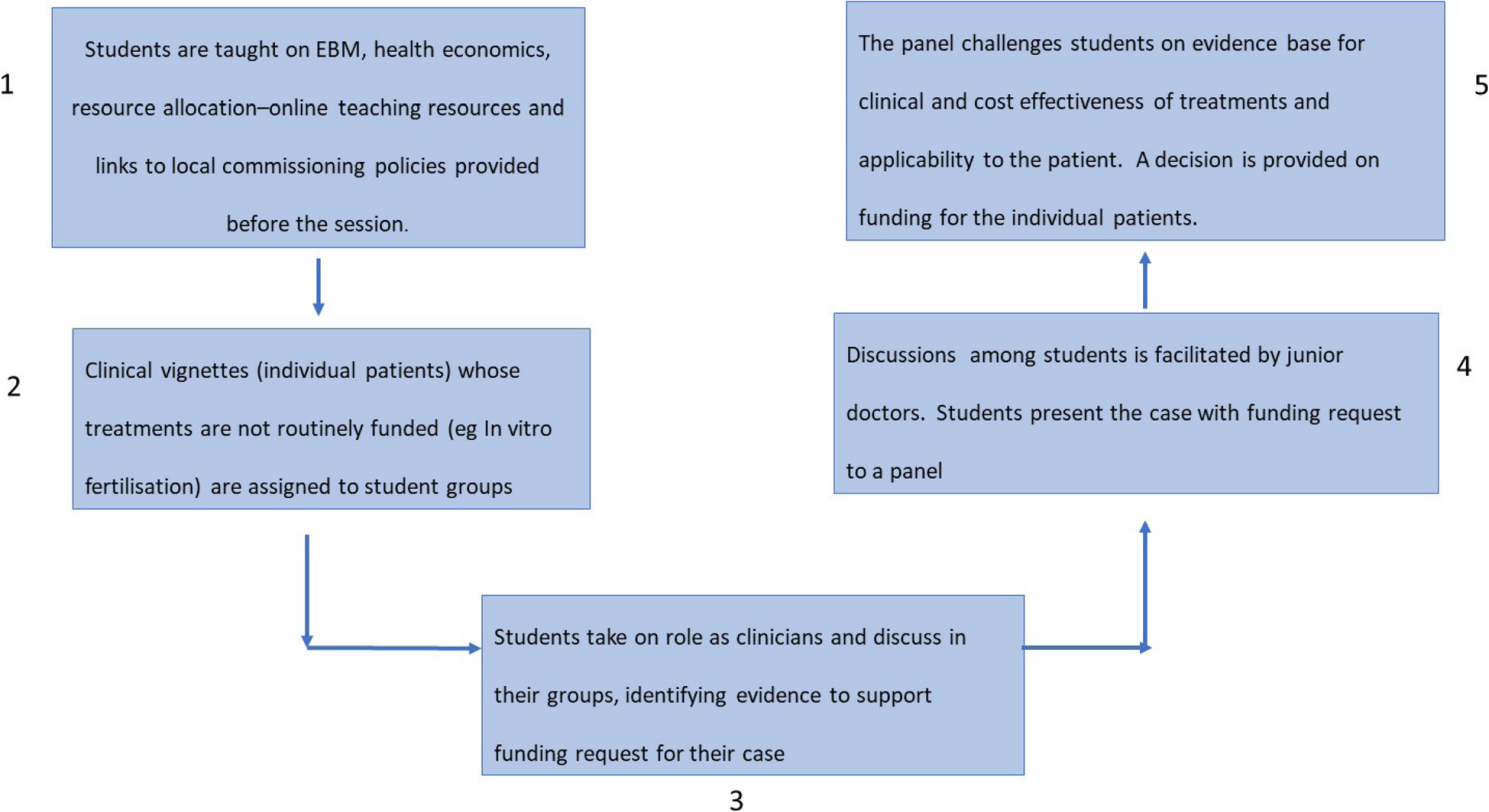
Flipped classroom model - an example used in second year EBM teaching

Flipped classroom methods have been introduced to ensure students receive an education tailored to their individual needs, providing flexibility and making the best use of technology which is desired by current undergraduate students [19, 20]. Students are instructed to do 1 to 3 hours of work in preparation for the flipped classroom session. The sessions start with a warmup activity such as a quiz competition between different student groups, followed by guided and independent practice. An example of a flipped classroom activity has been provided in Fig. 3.

### Design

This study has evaluated Phase I using a before-after study design. Data was gathered at baseline in year one before EBM teaching and at the end of year two EBM teaching (time points shown in Table 1). Ethical approval for the study was provided by the University of Buckingham School of Science, Medicine and Dentistry Ethics Committee. All students were invited to participate in the study and were introduced to the study purpose at the beginning of Phase I EBM teaching in term two. Informed written consent was obtained from participants at the start of the study.

The validated Fresno test [16] was used to assess the EBM competence of students before and after EBM teaching in Phase I. The Fresno test has been identified as a high quality tool as supported by established interrater reliability, use of objective (non-self-reported) outcome measure(s) and has demonstrated multiple (≥3) types of established validity evidence (including evidence of discriminative validity) [4, 15, 21]. The Fresno test has established content validity; an inter rater reliability of 0.76–0.98 for individual items; Cronbach’s alpha of 0.88; Item total correlation of 0.47–0.75; Item discrimination of 0.41–0.86 and construct validity- on the 212 point test, the novice mean was 95.6 and the expert mean was 147.5 (*p* < 0.001) [16]. The Fresno test is based on two clinical scenarios followed by 12 questions assessing competence in developing a focused clinical question, demonstrating literature searching skills, knowledge of study designs, critical appraisal of study quality and ability to calculate some basic statistics. Scores on the instrument range from 0 to 212. In this study, we used one clinical scenario at baseline and the second scenario at the end of the study. The test was administered online via the virtual learning environment (Moodle), along with a locally developed questionnaire. The questionnaire was designed based on a literature review of students’ perceptions of EBM and captured students’ self-reported knowledge and attitudes towards EBM. The questionnaire had five items - whether they had critically appraised journal articles before, their confidence in critically appraising a journal article, their ability to formulate a clinical question to start searching the best scientific evidence, perceptions of the relevance of EBM to patient care and relevance of EBM to their future practice as a doctor. The answer to the first question was reported as a ‘yes’ or ‘no’; while the answers to the remaining four questions were reported on a 5-point Likert scale ranging from strongly disagree to strongly agree.

### Participants

Participants were students who enrolled into the MBChB course in UBMS in 2017 (*n* = 83). A single cohort was invited to participate as we were exploring the changes in EBM competencies from baseline (before students had any EBM teaching) to the end of EBM teaching in year two of the MB ChB course. Of the 83 eligible students, 31 (36.4%) were recruited at baseline and 55 (64.7%) were recruited at end point. Eighteen students (21.7%) participated at both baseline and endpoint.

### Data collection

Students were invited to complete the Fresno test at baseline before EBM teaching. Completing the test at baseline was not compulsory and data from those students who gave informed consent was collected. At the end of EBM teaching in year two, the test was administered once again as a formative assessment and data were only used for this study if students gave written consent. Data collection points have been shown in Table 1.

### Statistical analysis

The data were anonymized and final scoring of the Fresno test was done by two faculty members independently using the Fresno marking rubric. The average of the scores from each rater were used in our analyses. The Inter-rater reliability between the two raters was assessed by calculating the intra class correlation (ICC). ICC estimates and their 95% confidence intervals were calculated based on a mean-rating (k = 2), consistency and 2-way mixed effects model (ICC 3,k), based on Koo and Li’s ICC selection algorithm [22]. The hypothesis that the average score would increase after EBM teaching was tested using a one-tailed, paired sample t-test. Statistical analyses were carried out using IBM SPSS version 20. We also used a box and whisker plot to visually display the distribution of scores at each time point, allowing comparison of the minimum, maximum, median and 1st and 3rd quartile scores. By comparing the distribution of scores in addition to the average scores, we could assess if any increase in average was due to an improvement in test performance by only a few students, or whether most students improved. For analyses of the questionnaire, we compared percentage of students’ responses in each category for all five questions before and after EBM teaching. We have used a stacked bar chart to present the data, as it can visually demonstrate any change in students’ self-reported knowledge and attitudes towards EBM.

## Results

Of the 83 eligible students, 31 (36.4%) completed the Fresno test at baseline and 55 (64.7%) completed the test at the end of the second year. Eighteen students completed the Fresno and questionnaire at both baseline and at the end of the second year. Data from these 18 students were used for the analyses of Fresno tests and the questionnaires. All the students were in the 18–25 age group range (Table 3). Six students were males (33%) and the remaining females (67%). Eight were undergraduate students (44%), while 10 already had an undergraduate degree (56%).

**Table 3:** Student characteristics

Characteristic		Sample for final data (*n* = 18)
Age	18–21	50% (*n* = 9)
	22–25	50% (*n* = 9)
Sex	Male	33% (*n* = 6)
	Female	67% (*n* = 12)
Undergraduate / Postgraduate	Undergraduate	44% (*n* = 8)
	Postgraduate	56% (*n* = 10)

Table 3 provides a breakdown of student characteristics.

### Students’ performance in the Fresno test

**Fig. 4:**
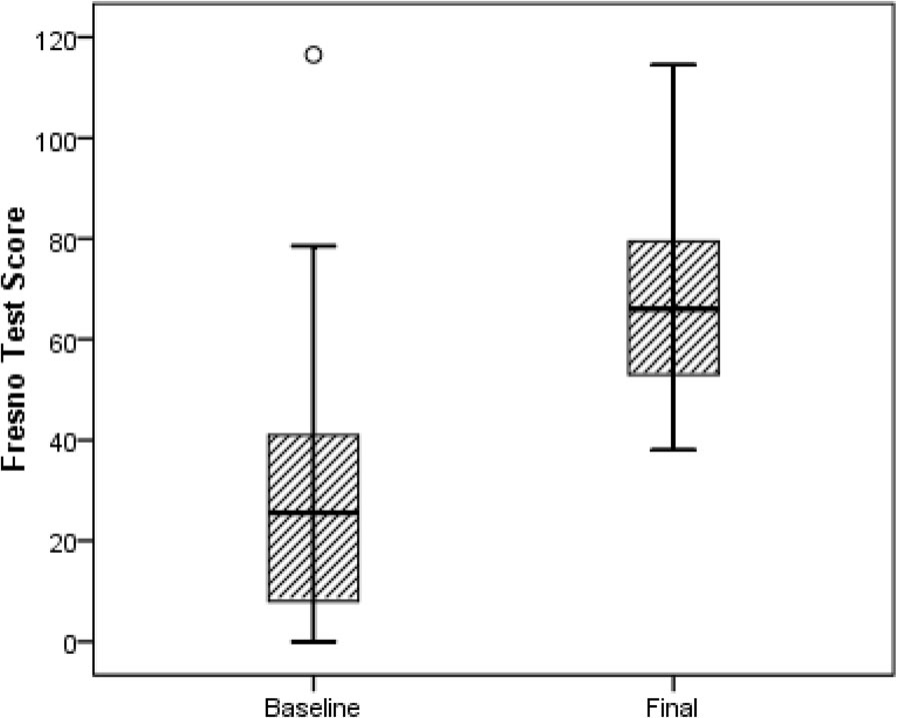
Comparison of performance data- distribution of all 18 students’ test scores at each time point

The distribution of all 18 students’ test scores at each time point is shown as a box and whisker plot (Fig. 4). At baseline, 50% of students scored 25.5 or above. After teaching, 50% of students scored 66.0 or above, an increase in the median of 35.3 marks. The minimum, maximum, 1st and 3rd quartile scores all also increased after teaching. The average score for the test was significantly higher after teaching than at baseline, with the average score increasing by 38.7 marks, from 29.3 at baseline to 68.0 after teaching (*p* < 0.001). The average scores for the whole test and each question at the two time points are shown in Table 4. There was excellent inter-rater reliability between the two Fresno test raters, with an ICC (3,k) of 0.97 (95%CI 0.92 to 0.99).

**Table 4:** Table of average scores, and change in average scores, for each question

Question	Question topic (maximum possible score)	Baseline	Final	Improvement	
		Average score	Average score	Change in score	1 tailed *p*-value
Q1	Asking a clinical question (24)	7.4	17.7	10.3	< 0.001*
Q2	Sources of evidence (24)	3.6	6.7	3.1	0.004*
Q3	Search strategy (24)	4.3	5.9	1.6	0.184
Q4	Study design (24)	3.2	12.2	9	< 0.001*
Q5	Relevance (24)	2	3.1	1.1	0.129
Q6	Internal validity (24)	4.6	6.5	1.9	0.174
Q7	Effect (24)	0.9	5.1	4.1	0.006*
Q8	Sensitivity, Specificity, PPV, NPV, likelihood ratio (20)	1.8	3.6	1.8	0.167
Q9	Absolute risk reduction, relative risk reduction, number needed to treat (12)	0.9	2.8	1.9	0.030*
Q10	Confidence interval (4)	0	1.6	1.6	0.002*
Q11	Best study design-diagnosis (4)	0	0.4	0.4	0.082
Q12	Best study design-prognosis	0.7	2.4	1.8	0.001*
Total	Total (212)	29.3	68	38.7	< 0.001*

*statistically significant

The average score was significantly higher after teaching for seven of the 12 questions. These seven questions assessed

- formulating PICO questions (Q1)
- information sources (Q2)
- study design (Q4)
- magnitude and significance (Q7)
- calculating risk and number needed to treat (Q9)
- confidence intervals (Q10)
- prognosis studies (Q12).

The largest increase in average score, 10.3 marks, was for the question on formulating PICO questions (Q1), despite students performing best on this question at baseline. The next largest increase in average score was for the question on study design (Q4), which increased by 9.0 marks. This has been reassuring as these are the key areas of focus in our Phase I EBM curriculum. Table 4 provides scores for each question at baseline and at the end of the study.

For the remaining questions, although there was an increase in average scores at follow-up, they were not statistically significant. These questions assessed search strategy (Q3); study relevance (Q5); study validity (Q6); calculating sensitivity, specificity, positive predictive value, negative predictive value, and likelihood ratio (Q8); and diagnostic studies (Q11). Although these topics are taught in Phase I, students seem to struggle with these concepts and interpreting these statistics. We will review our teaching methods for these topics in Phase I in addition to reinforcing them in Phase II of the MBChB course.

### Attitudes to EBM based on responses to the questionnaire

**Fig. 5:**
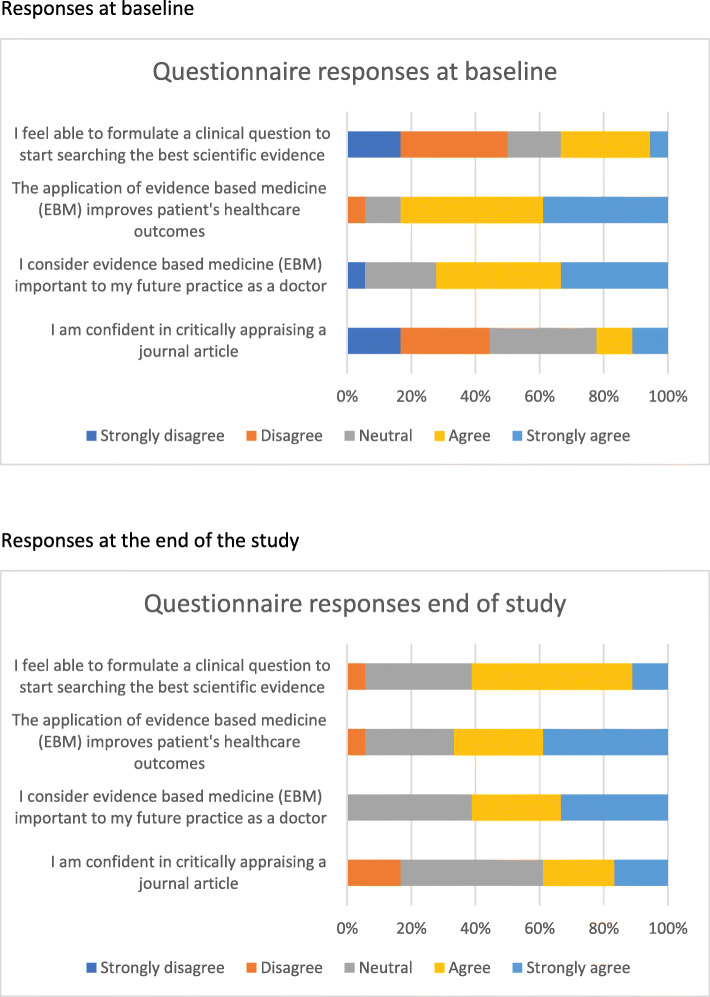
Students’ self-reported knowledge and attitudes towards EBM (before and after EBM teaching)

At the start of the course 33% (*n* = 6) of students had critically appraised journal articles and this increased to 89% (*n* = 16) at the end of EBM teaching. For the four questions where students had to choose on a Likert scale, the responses before and after EBM teaching are shown as a stacked bar chart in Fig. 5. At baseline, 50% (*n* = 9) felt they could not formulate clinical questions to start searching for the best evidence, however at the end only 5% (*n* = 1) still felt that way. At baseline, 44% (*n* = 8) felt they lacked confidence in critically appraising articles, while at the end of teaching, only 17% (*n* = 3) still felt that way. At baseline, except for one student, all students agreed that EBM was important to future practice as a doctor and that EBM improves patient outcomes. At the end, all students felt that EBM was important to future practice and all but one agreed that EBM improves patient outcomes.

## Discussion

This study has shown that it is feasible to introduce EBM as a multifaceted curriculum from the first year in an undergraduate medical school. Early evaluation of our curriculum in the first 2 years of MBChB has shown an increase in students’ knowledge, as assessed by their performance in the Fresno test, in addition to their self-rated EBM knowledge and self-reported attitudes about EBM.

Compared to baseline, at the end of EBM teaching, the average score for the Fresno test increased by 38.7 marks (*p* < 0.001). This finding is comparable with other studies evaluating longitudinal or integrated EBM teaching in a medical school setting [11, 13]. West et al. evaluated their longitudinal EBM curriculum in medical school and found that the knowledge scores in the Fresno test increased by 39.7 marks at the end of the second year portion of the course (*p* < 001) and by 54.6 marks at the end of the third year (*p* < 001) [11]. Aronoff et al. reported an average improvement of 11.1 +/− 20.0 marks in Fresno test scores in their study of an integrated EBM curriculum [13]. Aronoff et al. used an online didactic teaching model (web based) during clinical rotations, while ours was a blended teaching method with face to face didactic teaching, small group case-based discussions supplemented with online resources.

There was significant improvement in 7 out of 12 questions assessing different EBM skills. Knowledge appears to have improved following changes that have been made to the course and thus far have helped students gain a better understanding of EBM. The Fresno test, despite being time consuming for students and graders, has helped in assessing the effectiveness of our curriculum. Attitudes of students regarding the relevance of EBM to their future practice remained high throughout Phase I. Students felt more confident in critically appraising journal articles and in formulating clinical questions at the end of EBM teaching. Students reported that the curriculum had enhanced their understanding of the relevance of EBM to clinical practice.

The optimal time to introduce EBM in medical education has not been well researched yet. A study of junior doctor’s knowledge of and beliefs of EBM identified that while they agreed that EBM skills were essential to their clinical practice, they did not feel confident with their EBM skills as they did not have sufficient training [23]. Conversely, first year students who had not been exposed to the clinical environment could not appreciate the relevance of EBM to clinical practice [24]. This study has shown an early introduction to EBM in the first year of undergraduate medical program using multifaceted, teaching has been acceptable to students and has been effective in improving their EBM competency in the first 2 years. The study supports earlier research findings that delivering a series of educational interventions can promote reinforcement and development of student skills over a period and could result in significant improvements [25].

However, there are some limitations to the study. Firstly, there were no controls- whilst students served as their own controls, it is difficult to claim that the change was entirely due to the EBM teaching. However, designing a trial with ‘controls’ in medical education research can be quite challenging with students moving through educational processes in real time, experiencing rotations at different times and having the option to refuse randomization, yet cannot miss educational experiences [26]. Recruiting multiple sites can be quite expensive and pose an additional challenge of offering different training programs. The second limitation is that EBM is an important longitudinal theme in our curriculum and a significant component in all formative and summative assessments. Our findings may not be applicable to other settings where EBM has not been similarly integrated into the curriculum and assessments, though the study highlights the strength of such an integrated curriculum. Thirdly, though we invited the whole cohort to participate, only 18 students completed the Fresno test both before and after EBM teaching. The test took a long time to complete, which could explain the reluctance of students to attempt the test. Lastly, though the preliminary findings suggest improvement in knowledge and attitudes of students in Phase I of our curriculum, whether this will be reflected in students’ clinical practice can only be confirmed through longitudinal follow- up of these students when they are in clinical rotations and as junior doctors. Further research is needed to identify how our medical students integrate EBM knowledge and skills in real time clinical decisions.

To address some of the limitations of this study, the EBM knowledge and skills of the study cohort will continue to be assessed during Phase II of the curriculum. We are evaluating the effectiveness of our educational interventions using the Kirkpatrick’s four levels of evaluation-reaction, learning, behavior and results as our students progress along their undergraduate program [27]. In addition to the Fresno test, we will use data from the Assessing Competency in EBM (ACE) tool, which is shorter and easier to grade [28]. We will evaluate the effectiveness of our EBM curriculum by assessing our students’ ability to apply their EBM knowledge and skills in simulated or real time clinical scenarios using OSCEs, ACE and online educational prescriptions [29]. Both these tools have been shown to be feasible in assessing the application of EBM knowledge and skills in simulated or real clinical encounters. We will also seek students’ perceptions of the learning environment, integrating EBM teaching and assessments in clinical placements and identifying barriers and enablers in applying EBM in clinical practice.

## Conclusions

It has been feasible to design and integrate a multifaceted EBM curriculum in an undergraduate medical school starting in the first year of MBChB course. Early evaluation of the curriculum by assessing students’ competency using the validated Fresno test and their perceived knowledge towards EBM has shown to be effective. An early introduction to EBM principles to medical students before clinical placements and delivering a progressive curriculum with a series of educational interventions can promote reinforcement of EBM knowledge and skills.

## Data Availability

The data are available to all interested researchers upon request. Please contact the corresponding author.

## Abbreviations

EBM: Evidence Based Medicine
UBMS: University of Buckingham Medical School
OSCE: Objective Structured Clinical Examination
GMC: General Medical Council
ICC: Intra Class Correlation
PICO: Patient Intervention Control Outcome
ACE: Assessing Competency in EBM
